# OCA-MAC: A Cooperative TDMA-Based MAC Protocol for Vehicular Ad Hoc Networks

**DOI:** 10.3390/s19122691

**Published:** 2019-06-14

**Authors:** Yao Liu, Hongjing Zhou, Jiawei Huang

**Affiliations:** 1 School of Computer and Information Engineering, Hunan University of Technology and Business, Changsha 410205, China; yliu@hnuc.edu.cn (Y.L.); hjzhou@hnuc.edu.cn (H.Z.); 2 Key Laboratory of Hunan Province for Mobile Business Intelligence, Hunan University of Technology and Business, Changsha 410205, China; 3 Mobile E-Business Collaborative Innovation Center of Hunan Province, Hunan University of Technology and Business, Changsha 410205, China; 4 School of Computer Science and Engineering, Central South University, Changsha 410083, China

**Keywords:** vehicular networks, medium access control, time division multiple access, cooperative communication

## Abstract

Cooperative communication is an effective method of improving the transmission performance for vehicular ad hoc networks. However, the rapid movement of vehicles leads to frequent changes in network topology and reduces the probability of successful data transmission on the medium access control (MAC) layer. In this paper, we propose an Optimal Cooperative Ad hoc MAC protocol (OCA-MAC) based on time division multiple access (TDMA). OCA-MAC utilizes multiple cooperative nodes to forward data, so as to improve the probability of successful data transmission. It chooses cooperative nodes according to direct successful transmission probability, communication range between potential helper node and destination node, and available time slot. Meanwhile, in order to avoid excessive transmission redundancy caused by multiple cooperative forwarding, the optimal number of cooperative forwarding nodes is obtained through analysis of a probabilistic model. Simulation results show that OCA-MAC improves the successful data transmission rate and reduces the number of transmission times and transmission delay compared to the multichannel TDMA MAC protocol (VeMAC) and the cooperative ad hoc MAC protocol (CAH-MAC).

## 1. Introduction

A growing number of new applications, such as traffic accidents warning, assistant driving, special information query, multimedia information dissemination, and data collecting for smart cities, are emerging in the vehicular ad hoc networks (VANETs) [1,2,3,4]. VANETs are particular kinds of mobile ad hoc networks (MANETs), and they are important components of intelligent transportation systems (ITSs). ITSs can provide a lot of convenient and safe service when people are driving. Vehicles can access to the data center backbone networks [5,6] through infrastructure on the road side for traffic conditions. Avoiding traffic congestion and reducing access delay need to be carefully considered [7,8]. Drivers could react to accidental situations on roads in advance. In VANETs, special devices need to be installed for communication. On-board units (OBUs) are equipped on vehicles, and road-side units (RSUs) are deployed on the roadsides. VANETs support vehicle-to-vehicle (V2V) and vehicle-to-infrastructure (V2I) communication methods to transmit various kinds of messages. VANETs exhibit unique characteristics, which are different from those in general MANETs, for dynamic topology, high mobility, and ample computing and storage capacities.

In VANETs, vehicles can organize in an ad hoc manner and forward messages hop by hop. For the V2V communication method, there is no central communication coordinator, and multiple nodes share a wireless channel. Transmission collisions frequently occur when many applications broadcast information to adjacent vehicles. Therefore, a medium access control (MAC) protocol is vital for guaranteeing quality of service (QoS) in VANETs. In order to support QoS in VANETs, the MAC protocol needs to provide reliable communications and efficiently utilize the shared wireless channel. Traditional wireless MAC protocols are inappropriate for VANETs. Because nodes use a shared wireless channel by using the same radio frequency in VANETs, inappropriate MAC protocols may result in collisions among nodes, and much of the bandwidth of wireless channel will be wasted. Hence, reliable multi-hop communication is a crucial issue in VANETs, and efficient approaches for increasing radio resources utilization should be designed.

Due to the considerations of cost and availability, CSMA-based MAC, for example, IEEE 802.11p is used for VANETs. IEEE 802.11p cannot handle the hidden terminal problem for its lack of RTS/CTS mechanism. It does not provide a reliable broadcast service. In the situation of high vehicular traffic density, wireless channel will suffer from great congestion. Therefore, the bandwidth of channel is wasted. If multiple channels are used, VANETs have to face the multi-channel synchronization and co-channel interference problems. Clearly, QoS cannot be supported by MAC protocols based on IEEE 802.11 with distributed coordination function.

In order to guarantee QoS in the MAC layer, various MAC protocols, such as time division multiple access (TDMA)-based, space division multiple access (SDMA)-based, and code division multiple access (CDMA)-based, have been presented in recent years. Reservation-based MACs are widely adopted for wireless networks. Because of dynamic topology changes, TDMA-based MAC protocols may result in waste of time slots. Cooperative communication for MAC protocols has great advantages in enhancing the overall performance of wireless networks [9]. Cooperative communication is a powerful and effective method to enhance performance in multi-hop wireless systems. Reliable cooperative communication in VANETs relies on the status of the surrounding vehicles.

In this paper, we propose an Optimal Cooperative Ad hoc MAC protocol, called OCA-MAC, based on TDMA for VANETs. We designed the method of choosing the optimal cooperative node and improved the successful transmission rate. Meanwhile, the optimal number of cooperative nodes was obtained by analysis of a probabilistic model, which can avoid redundant transmission overhead caused by excessive cooperative forwarding. The main contributions of this paper are list as follows:

- We propose a TDMA-based MAC protocol to improve the successful transmission rate for VANETs, which uses cooperative nodes to forward data;
- We established an effective probabilistic model for cooperative communication and obtain the optimal value of the number of cooperative nodes under different channel;
- We present extensive simulation results to verify the throughput of the proposed protocol and show that the proposed protocol can achieve a successful transmission rate and reduce the number of transmissions and transmission time compared to the multichannel TDMA MAC protocol (VeMAC) and the cooperative ad hoc MAC protocol (CAH-MAC).

The remaining paper is organized as follows. Section 2 reviews the previous work on MAC protocols in VANETs. Section 3 presents the OCA-MAC protocol. Section 4 analyzes the performance of the OCA-MAC protocol. In Section 5, the OCA-MAC protocol is evaluated with simulations. Finally, Section 6 concludes this paper.

## 2. Related Work

So far, a number of MAC protocols have been proposed to deal with the problem of transmission for VANETs. The existing vehicular MAC protocols are usually classified into three categories, namely, contention-based protocols, contention-free protocols, and hybrid protocols [10].

Contention-based MAC protocols are random access protocols. Vehicles can randomly access the shared wireless channel when they want to send data. In VANETs, the IEEE 802.11p [11] is used for medium access control. It is a distributed random channel access protocol based on carrier sense multiple access with collision avoidance (CSMA/CA). It addresses channel collisions using binary exponential back-off. The main challenge is frequent message collisions, especially in the situation of high traffic loads. The IEEE 802.11p cannot guarantee the upper latency, and then it is not suited for time-sensitive applications [12].

In order to improve transmission efficiency in wireless networks, distributed cooperative MAC protocols based on IEEE 802.11 [13,14,15,16,17,18] are proposed. The key factor of transmission efficiency is the choice of cooperative nodes. In [13,14], it was pointed out that nodes could decrease the sending rate for the weak signal. The node with a high sending rate will be selected as the third party cooperative node. The network throughput is improved by changing the original one-hop low-rate link to the two-hop high-rate link. A novel coordinated cooperative MAC protocol (CCMAC) [15] can support parallel transmission among nodes. This method can reduce congestion at the access point and thus enhance network throughput. The MAC protocols mentioned above choose cooperative nodes according to the history transmission information. In VANETs, nodes move fast, and topology changes dynamically. Therefore, the history information cannot reflect the current status of the channel. The choice of relaying nodes based on history information is not adapted in VANETs [16]. In [17,18], novel protocols were present which can determine the cooperative node. The cooperative diversity MAC protocol (CD-MAC) [17] chooses the node that has the strongest signal as the relaying node. When the source node fails to transmit data to the destination node, the relaying node will forward the data. Zhang et al. [19] proposed a vehicular cooperative MAC protocol (VC-MAC) to maximize system throughput by utilizing spatial reusability. In dense vehicular network scenarios, VC-MAC will face a serious problem of exposed nodes, which results in high channel access delay. In order to improve network throughput, two-cycle cooperative MAC protocol (VC2-MAC) [18] uses the cooperative node to retransmit data when the node fails to receive data from RSUs. VC2-MAC can also reduce the channel access delay. The cooperative MAC protocols based on IEEE 802.11 have the following problems. The cooperative nodes will not forward their own data until the cooperation is ended. Because cooperative nodes join the data forwarding, the probability of generating hidden and exposed nodes is further increased.

Contention-free protocols allow nodes to share wireless channels according to different pre-assigned metrics, such as time slot, frequency, and code. In contention-free protocols, the intermediate node uses its free time slots to forward packets. The MAC protocols based on TDMA, such as ADHOC MAC [20], VeMAC [21], self-organizing time division multiple access protocol (STDMAC) [22], collision free reservation MAC protocol (CFR MAC) [23], distributed and infrastructure free TDMA based MAC protocol (DTMAC) [24], and multi-channel MAC protocol (MCMAC) [25], are proposed to facilitate a reliable delay. Each node sends data in a dedicated time slot during each frame time. ADHOC MAC chooses a node which is in the range of communication as the relaying node. ADHOC MAC is not adaptive to the changing traffic because of using a fixed frame length. However, VeMAC chooses the nodes from the opposite direction. It means VeMAC selects the farthest node as the relaying node. The successful transmission rate decreases sharply for the reason of topology change, signal attenuation, and broadcast interference, which are caused by fast mobility. The limited time slots are wasted, and the transmission efficiency in the network is reduced. STDMA is designed for real-time applications and performs well in terms of fairness. CFR MAC can alleviate the mobile hidden terminal problem and solve collisions caused by high moving speed. It is also suitable for real-time communications. DTMAC uses the linear feature of network topology and the location information to help vehicles to reduce access collisions and merging collisions. DTMAC needs to dissect the road into small fixed areas.

Cooperative communication has also been proposed for the TDMA-based MAC protocols to improve transmission efficiency and reliability [26,27]. The communication environment is composed of a central controller and mobile nodes. In the communication process, cooperative transmissions among the fixed auxiliary nodes are coordinated by the central controller. In [27], the time slot of the cooperative node was fixed, and it will not be occupied by other nodes for data transmission even if no cooperation occurs. Because of the limitations of these protocols themselves and the characteristics of vehicular networks, the above protocols cannot be directly applied to vehicular networks. In order to adapt the situation of fast moving nodes, Bharati et al. proposed a cooperative distributed TDMA-based MAC protocol, CAH-HAC [28]. CAH-MAC does not rely on infrastructure, and it allows each node to share wireless channels for other nodes in the unreserved time slots. The sending node randomly selects a neighbor as the relaying node. It uses the free time slot to forward data and improves the successful transmission rate. However, it cannot provide the reliability of cooperative forwarding.

In hybrid TDMA/CSMA multichannel MAC protocols, collisions will occur when two or more vehicles attempt to use the same free time slot. Santos et al. proposed a reconfigurable and adaptive TDMA protocol (RA-TDMA) [29]. RA-TDMA constructs an overlay for collaborative applications using TDMA round. The CSMA/CA mechanism is still used to tolerate external traffic. Zhang et al. [9] proposed a jamming signal-based time acquisition scheme to improve the efficiency of time slot acquisition. The hybrid efficient and reliable MAC protocol (HER-MAC) uses both CSMA and TDMA schemes [30]. Nguyen et al. proposed a hybrid TDMA/CSMA multi-channel MAC protocol (HTC-MAC) [31] based on HER-MAC. HTC-MAC increases the throughput and reduces unnecessary control overhead on the control channel. Dan et al. proposed a cooperative–efficient–reliable MAC protocol (CER-MAC) [32]. CER-MAC allows nodes to exploit their own pre-allocated time slots to transmit messages. Nodes can also use neighbor’s reserved time slots. Nodes store safety messages in their buffers and broadcast twice to achieve reliability. Although hybrid cooperative MAC protocols take advantage of CSMA/CA and TDMA protocols, they bring complexity of implementation and produce more unnecessary control messages.

## 3. Protocol Design

In this section, we first describe the basic idea in a typical VANET scenario. Next, we present the cooperation conditions among nodes. Then, we illustrate the process of cooperative forwarding in detail. Finally, we discuss how to select the optimal cooperative node.

### 3.1. Motivation

**Figure 1:**
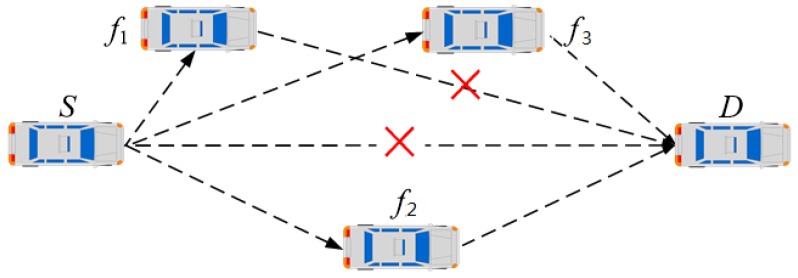
Vehicular ad hoc network (VANET) scenario on the highway.

A typical VANET scenario is depicted in Figure 1. Vehicle *S* is the source node, and vehicle *D* is the destination node. All the vehicles have the same communication range *r*. Vehicles, including *f${}_{1}$*, *f${}_{2}$*, *f${}_{3}$*, and *D*, are in the communication range of source node *S*. The destination node *D* cannot receive data from the source node *S* for reasons of channel fading or mobility. The other vehicles, namely, *f${}_{1}$*, *f${}_{2}$*, and *f${}_{3}$*, are located between the source node *S* and the destination node *D*. Therefore, they become potential cooperative relaying nodes. In this case, vehicle *f${}_{1}$* cannot forward data to vehicle *D* yet. Vehicle *f${}_{2}$* is located in the middle place between the source node and the destination node, and then it is the ideal cooperative node. In order to promote the successful transmission rate, vehicle *f${}_{3}$* can also be selected as a cooperative node to forward data to the destination node.

Obviously, the efficiency of data transmission is affected by the channel condition. The probability of a successful receiving rate can be improved by designing a proper cooperative forwarding scheme. In the following subsections, we illustrate the design of a cooperative TDMA-based MAC protocol. It contains the conditions of joining the cooperative forwarding, the cooperative procedure, and the method of determining the optimal node.

### 3.2. Cooperative Condition

Cooperative node is the common neighbor node of the source node and the destination node. It can forward the source node’s data repeatedly in the free time slot. Therefore, the reliability of data reception by the destination node can be improved in the case of bad channel conditions. If a node satisfies the following three conditions, it can be selected as a cooperative node:

(1) The successful transmission rate is lower than a predetermined threshold. According to the vehicle density, history transmission information, and the distance to the destination node, the source node will prejudge the probability of successful transmission before transmission. If the successful transmission rate is below the predefined threshold, the cooperative node forwarding mechanism is initiated;
(2) Cooperation is only executed when the destination node is in the communication range;
(3) There must be at least one available time slot, and the cooperative node can transmit the data.

### 3.3. Cooperative Forwarding

Each node sends its control frame periodically. The required control information is added into the control frame. The control frame contains the current node ID, the occupied time slot number, one hop neighbors ID, and their occupied time slot number. Every node maintains the content of the neighbor list by exchanging the control frame. The content includes the number of the entire one hop and two hop neighbor nodes and the number of time slots occupied by the nodes. Meanwhile, the ratio of successfully receiving the neighbor’s control frame is used to evaluate the transmission quality of channel.

When the sending node wants to send data, a node between the sending node and the target node is selected as the default forwarding node. Meanwhile, the sending node calculates the successful transmission rate between the target node and itself according to the historical information. The cooperation will start if the successful transmission rate is lower than the preset threshold value. Specifically, the sending node adds a cooperation head to the packet. The header information includes the ID of potential cooperative nodes and the number of free time slots to be allocated. At the same time, in order to avoid too much cooperative transmission overhead, the sending node will set the number of cooperative nodes. The specific method of selecting cooperative nodes and calculating the number of cooperative nodes, *K*, is discussed in detail in Section 3.4 and Section 4, respectively. Then, the potential cooperative nodes monitor the channel and count the number of packet transmission when they receive the data packets with the cooperation header. If the number of the data packets sent by other cooperative nodes is less than *K*, the data packets are forwarded by the current node in the allocated free time slot.

**Figure 2:**
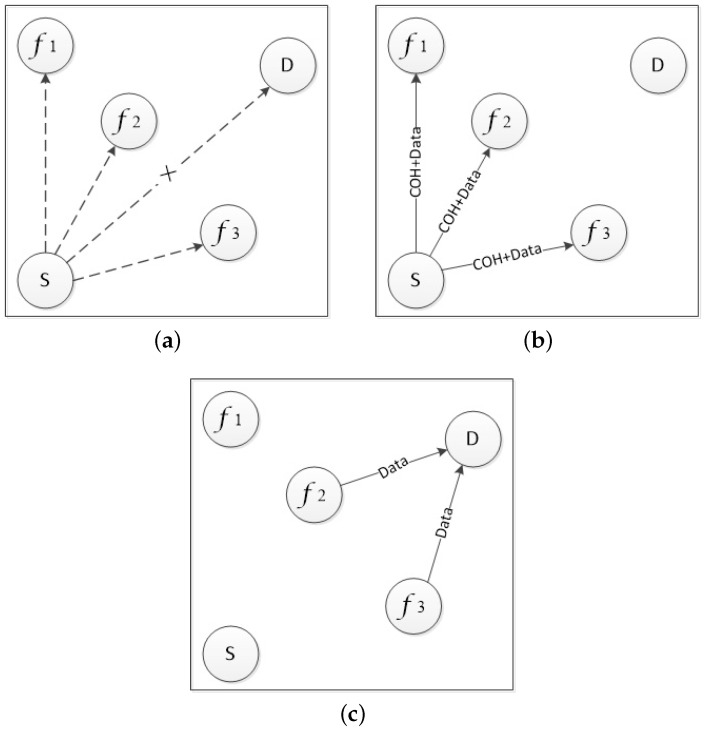
The procedure of information exchanges in optimal cooperative ad hoc-medium access control (OCA-MAC). (**a**) Original status. (**b**) Potential cooperative nodes selection. (**c**) Cooperative forwarding.

Figure 2 shows a typical cooperative process of the OCA-MAC protocol. When the sending node *S* determines its successful transmission rate of sending data to the receiving node *D* is below the predesigned value, node *S* will choose *f${}_{1}$*, *f${}_{2}$*, and *f${}_{3}$* as potential cooperative nodes. We assume that the number of cooperation nodes is two in this scenario. When *f${}_{1}$*, *f${}_{2}$*, and *f${}_{3}$* receive the packet with the cooperation header, they participate in the cooperative forwarding. In order to avoid excessive transmission overhead caused by redundant transmission, node *f${}_{1}$* will give up forwarding if it detects the number of forwarding reaches the predesigned number *K*. If the receiving node *D* receives the redundant packets, it will drop the packet and avoid invalid overhead.

### 3.4. Selection of the Optimal Cooperative Node

In order to improve the successful rate of cooperative forwarding, we designed a method of choosing the optimal cooperative node. We assumed that the successful transmission rate *P*${}_{s}$ can be simplified as a function related to the communication range. The successful transmission rate decreases when the distance between the sender and the receiver becomes larger. In such conditions, OCA-MAC will select the nodes which are located near the midpoint between the source node and the destination node as the relaying node in priority order. The forwarding successful rate through the relaying node depends on the successful forwarding rate of the separate links. Obviously, the total successful forwarding rate reaches the maximum when the successful forwarding rates of both links are equal.

Therefore, the sending node *S* will calculate the distance between the position of potential cooperative nodes and the midpoint between the sending node and the receiving node according to Equation (1):

(1) $$d_{n}=\sqrt{{\left(x_{n}-\frac{x_{s}+x_{d}}{2}\right)}^{2}+{\left(y_{n}-\frac{y_{s}+y_{d}}{2}\right)}^{2}},$$

where (*x*${}_{n}$, *y*${}_{n}$), (*x*${}_{s}$, *y*${}_{s}$), and (*x*${}_{d}$, *y*${}_{d}$) are the coordinates of the potential relaying node, the source node, and the destination node. Then, the free time slots are allocated to the potential nodes in ascending order of the value *d*${}_{n}$. The node that has the minimal *d*${}_{n}$ is allocated the top-ranked free time slot.

In the process of choosing the cooperative nodes, another important issue is to determine the number of cooperative nodes. Under bad channel conditions, the reliable data forwarding successful rate cannot be guaranteed with a lower number of nodes. Under good channel conditions, too many cooperative nodes can cause excessive transmission overhead. The effective throughput is decreased. In the next section, we analyze how to determine the optimal number of cooperative nodes under different channel conditions.

## 4. Analysis of the Number of Cooperative Nodes

In this section, we first illustrate a mathematical model in a cooperation network for evaluating effective throughput. Then, we acquire the optimal number of cooperative nodes in different channels.

We assume that vehicles are distributed randomly on the lane, and the number of nodes follows a Poisson distribution. Let β be the vehicle density and *r* be the communication range. The probability of finding *i* vehicles in a communication range *r* is given by:

(2) $$p\left(i,2r\right)=\frac{{\left(2\beta r\right)}^{i}e^{-2\beta r}}{i!}.$$

There are three types of time slots in a frame, namely, free time slots, successful time slots, and failed time slots. The free time slot, τ${}^{free}$, refers to the time slot which is not used by any nodes. A time slot in which data are successfully delivered to the receiving node is said to be a successful time slot, τ${}^{succ}$. The failed time slot, τ${}^{fail}$, refers to the time slot when data transmission is unsuccessful. Let random variables *F*${}_{free}$, *F*${}_{succ}$, and *F*${}_{fail}$ represent the number of free time slots, the number of successful time slots, and the number of failed time slots, respectively.

In the process of cooperative transmission, the sending node *S* will forward data in the free time slot when it fails to send data to the receiving node directly. If the data are successfully received by the destination node *D* through the help of the cooperative node, the free time slot turns to the successful time slot. Otherwise, it becomes a failed time slot. Because OCA-MAC adopts the TDMA scheme, in which time slots are allocated in advance, there is no channel collision. The successful transmission rate *P*${}_{s}$ is only related to the quality of the wireless channel. When the number of free time slots *F*${}_{free}$ is *n*, the number of successful slots *F*${}_{succ}$ follows binomial distribution:

(3) $$\mathrm{Pr}\left\{F_{succ}=m|F_{free}=n\right\}=C_{F-n}^{m}{\left(P_{s}\right)}^{m}{\left(1-P_{s}\right)}^{F-n-m},m=0,1,\cdots ,F-n.$$

The expectation of *X* is:

(4) $$E\left(F_{succ}|F_{free}=n\right)=\left(F-n\right)P_{s}.$$

A frame contains *F* time slots, and each node will occupy a time slot. The number of free time slots *U* can be expressed as:

(5) $$F_{free}=\left\{\begin{array}{c} 0,\;\;\;i\geq F\\ F-i,\;\;1\leq i<F \end{array}.\right.$$

From Equations (2) and (5), we can get the probability of a free time slot.

(6) $$\mathrm{Pr}\left\{F_{free}=n\right\}=\left\{\begin{array}{c} 1-\sum_{m=1}^{F-1}\frac{{\left(2\beta r\right)}^{m}e^{-2\beta r}}{m!},\;n=0\\ \frac{{\left(2\beta r\right)}^{F-n}e^{-2\beta r}}{(F-n)!},\;\;\;\;\;0<n\leq F-1 \end{array}\right.$$

From Equations (4) and (6), we can get the expected value *E(F${}_{succ}$)* of a successful time slot.

(7) $$E\left(F_{succ}\right)=P_{s}F\left(1-\sum_{n=1}^{F-1}\frac{{\left(2\beta r\right)}^{n}e^{-2\beta r}}{n!}\right)+P_{s}\left(F-n\right)\sum_{n=1}^{F-1}\frac{{\left(2\beta r\right)}^{F-n}e^{-2\beta r}}{(F-n)!}$$

In the OCA-MAC protocol, cooperation will yield benefits when the cooperative nodes successfully delivery data to the destination node. We assume the number of cooperative nodes is *n${}_{c}$*. The probability *P*${}_{coop}$ that at least one cooperative node succeeds in forwarding data to the destination node is:

(8) $$P_{coop}=1-{\left(1-P_{s}\right)}^{n_{c}}.$$

The total successful transmission rate *P${}_{s\_coop}$* in the cooperative model is:

(9) $$P_{s\_coop}=P_{s}+\left(1-P_{s}\right)P_{coop}.$$

Therefore, the expected value, *E(F${}_{coop}$)*, of the successful time slot can be expressed as:

(10) $$E\left(F_{coop}\right)=P_{s\_coop}F\left(1-\sum_{n=1}^{F-1}\frac{{\left(2\beta r\right)}^{n}e^{-2\beta r}}{n!}\right)+P_{s\_coop}\left(F-n\right)\sum_{n=1}^{F-1}\frac{{\left(2\beta r\right)}^{F-n}e^{-2\beta r}}{(F-n)!}.$$

When the cooperative nodes participate in the forwarding, the successful acceptance rate of the destination node is enhanced. The throughput of the whole network is also reduced because the redundant data are generated by the cooperative nodes. Throughput is defined as the ratio of successful time slots to the total number of time slots in each frame. The actual network throughput of the cooperative mechanism in the OCA-MAC protocol *G*${}_{coop}$ is:

(11) $$G_{coop}=\frac{E\left(F_{coop}\right)}{F}\times \frac{F-n_{c}}{F}=\frac{E\left(F_{coop}\right)\left(F-n_{c}\right)}{F^{2}}.$$

We consider the relation between the number of cooperative nodes and *G*${}_{coop}$ in the case of maximum *G*${}_{coop}$. We derive *G*${}_{coop}$ from *n${}_{c}$*. When the derivative equals 0, we can get the value of *n${}_{c}$*, which maximizes the *G*${}_{coop}$ value. The expression of *n${}_{c}$* is as follows:

(12) $$n_{c}=\frac{\left(lambertW\left(0,-\exp\left(1-F*\log\left(1-P_{s}\right)\right)/\left(P_{s}-1\right)\right)+F*\log\left(1-P_{s}\right)-1\right)}{\log(1-P_{s})}.$$

**Figure 3:**
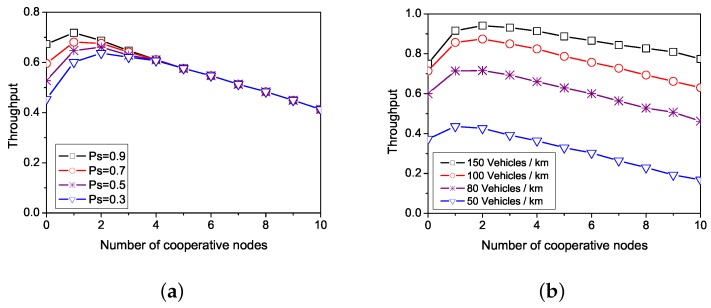
Network throughput. (**a**) Influence of successful transmission rate on throughput. (**b**) Influence of node density on throughput.

Figure 3 shows the network throughput with a different number of cooperative nodes when the number of time slots *F* is 60. Figure 3a shows the network throughput with a vehicle density of 75 vehicles per kilometer. It can be seen that the higher the successful rate of channel transmission, the higher the effective throughput of the network. Figure 3b shows the network throughput when the channel successful transmission rate is 0.75. With the increase of vehicle density, the growing numbers of vehicles participate in communication. Then, the channel time slot is fully utilized, and the network throughput is also increased.

At the same time, we can see from Figure 3 that the network throughput increases in the beginning with the number of cooperative nodes increasing. The cooperative nodes are able to help with forwarding data to the destination node and improving throughput. When excessive cooperative nodes participate in the forwarding, they will generate channel redundancy and lead to decreasing the total throughput after reaching the highest value. As a whole, the total network throughput has the highest value when the number of cooperative nodes is between 1 and 3 in the condition of different channel states and node density. Therefore, we set the number of cooperative nodes to 2 in the OCA-MAC protocol.

## 5. Performance Evaluation

To evaluate the performance of different MAC protocols, we implemented VeMAC, CAH-MAC, and OCA-MAC using an NS2 simulator. VeMAC is a MAC protocol based on TDMA. It chooses the farthest node in the communication range as a relay node. CAH-MAC is also a TDMA-based MAC protocol. In order to improve transmission efficiency, it selects a random cooperative node to forward data.

The performance metrics that are used to compare different MAC protocols contain transmission delay, number of transmissions, and successful transmission rate. Transmission delay is the latency from the source node to the destination node along the path. The number of transmissions refers to the total end-to-end transmission numbers of all the nodes. It also includes both the successful and failed transmission numbers. The successful transmission rate is defined as the ratio of the successful transmission numbers to the total transmission numbers.

The whole simulation scenario is on the highway. The simulation parameters are summarized in Table 1. The experimental results are the average values of 100 simulation runs. The successful transmission rate of node *P*${}_{s}$ is defined as *P*${}_{s}=1-d^{2}/r^{2}$ , where *d* is the distance between the sending node and the receiving node, and *r* is the transmission range of the node.

### 5.1. Performance Distribution of All the Nodes

In this subsection, we consider the common scenario of a segment of a vehicle traffic road with 50 vehicles. Each vehicle moves in the same direction with a constant speed. The number of vehicles does not change during the simulation time. A vehicle can communicate with other vehicles within its transmission range. The source node is at the tail of the segment, and the destination node is at the head of the segment. From the tail to the head, the ID of each vehicle is labeled from 1 to 50 in turn. The source node ID is 1, and the destination node ID is 50.

**Figure 4:**
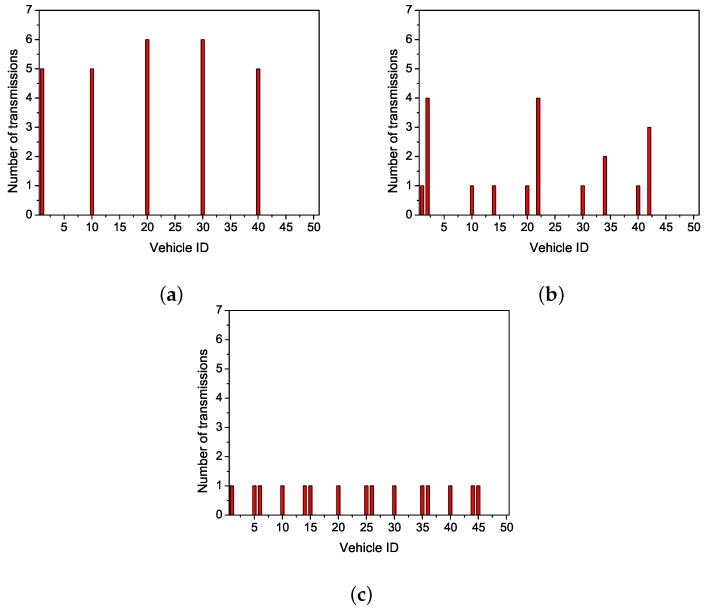
Comparisons of the number of transmissions. (**a**) multichannel TDMA MAC protocol (VeMAC). (**b**) cooperative ad hoc MAC protocol (CAH-MAC). (**c**) OCA-MAC.

First, we tested the number of transmissions of all nodes. Figure 4a shows that every forwarding node has a higher number of transmissions, but the number of forwarding nodes is small. VeMAC chooses the farthest node within the scope of current communication range to forward data. The long distance results in the decrease of the successful transmission rate and the increase of the number of retransmissions. Thus, the total number of transmission increases. Figure 4b shows that more nodes participate in data forwarding in CAH-MAC. That is because CAH-MAC makes use of cooperative nodes to forward data. Accordingly, the successful transmission rate improves, and the number of retransmissions decreases. Owing to a random choice of cooperative nodes, there will exist a higher number of retransmissions in cooperative transmission occasionally. For example, the 2nd node and the 22nd node in Figure 4b. Figure 4c shows that OCA-MAC has more nodes than CAH-MAC participating in forwarding. In OCA-MAC, nodes have the lowest forwarding times as well. OCA-MAC makes the optimal choice for cooperative nodes on the basis of CAH-MAC, which avoids retransmission due to the unreasonable selection of cooperative nodes.

**Figure 5:**
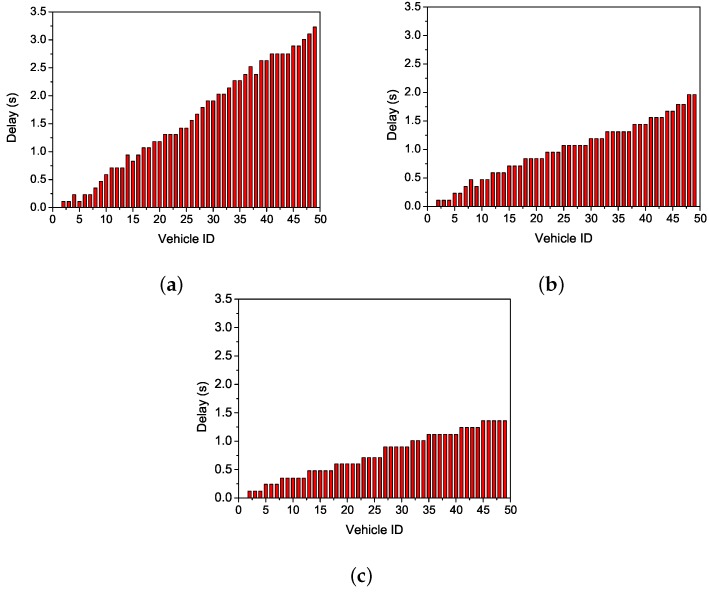
Comparisons of delay. (**a**) VeMAC. (**b**) CAH-MAC. (**c**) OCA-MAC.

Next, we compared the packet receiving delay of each node from the source in three protocols. Figure 5 shows that OCA-MAC performed better than the other two protocols in terms of delay. OCA-MAC has the shortest packet delivery time, which is about 26% and 50% lower than those of CAH-MAC and VeMAC, respectively. The reason is that the VeMAC protocol has the highest number of retransmissions, which results in more transmission time. Compared to CAH-MAC, OCA-MAC has an advantage in choosing cooperative nodes. OCA-MAC has a lower number of transmissions than CAH-MAC. Thus, it has the shortest transmission time.

### 5.2. Influence of the Number of Nodes

**Figure 6:**
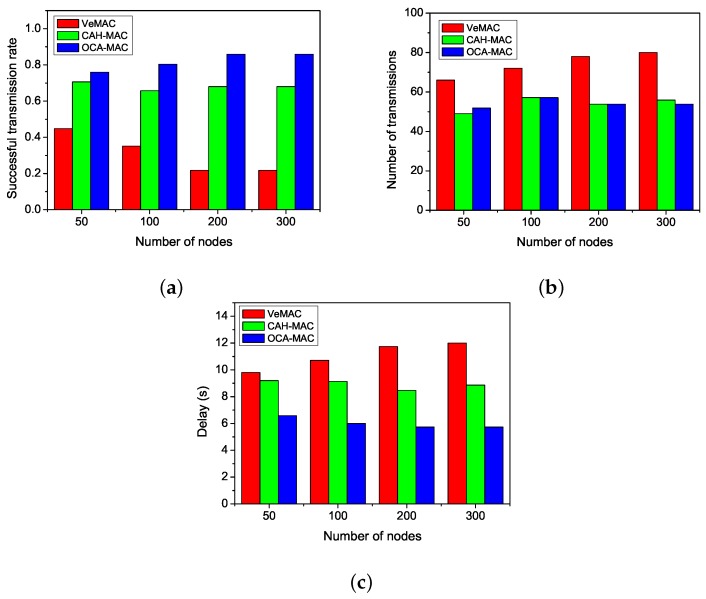
Influence of the number of nodes on the performance. (**a**) Successful transmission rate. (**b**) Number of transmissions. (**c**) Delay.

In this experiment, the number of nodes varies from 50 to 300 per kilometer. Figure 6 shows the influence of the number of nodes on the performance. It can be observed that OCA-MAC has a better performance than the other protocols. OCA-MAC can find enough cooperative nodes when the number of nodes increases to some extent. If the number of nodes continues to increase, the performance improvement of OCA-MAC is not apparent. The scenario of 300 nodes represents that there are enough potential nodes for cooperative transmission.

We can learn from Figure 6a that the successful transmission rate of OCA-MAC is nearly 300% higher than that of VeMAC and about 20% higher than that of CAH-MAC in the situation of 300 nodes. When the number of nodes is small, the gap between OCA-MAC and CAH-MAC is small. As the number of nodes grows, the number of available cooperative nodes increases. The successful transmission rate of CAH-MAC does not vary obviously due to the random choice of cooperative nodes. Meanwhile, OCA-MAC has more opportunities to choose better cooperative nodes. It can promote the successful transmission rate to a certain extent.

It can be seen from Figure 6b that the number of transmissions of VeMAC increases when the number of nodes increases. The main reason for the increase of transmission numbers of VeMAC is that the access collisions increase gradually. However, the number of transmissions of both CAH-MAC and OCA-MAC maintains relative stability, and there is no significant difference of transmission numbers between OCA-MAC and CAH-MAC. OCA-MAC decreases the number of transmissions about 30% compared to VeMAC when the number of nodes is 300. Although the number of nodes increases, more nodes can provide transmission opportunities. Due to the limitation of the number of cooperative nodes, the number of nodes that are really used for cooperative transmission does not change sharply. Therefore, the number of transmission of CAH-MAC and OCA-MAC will not have great changes in this case.

Figure 6c shows that the delay of VeMAC also increases with the number of nodes. In VeMAC, the increasing number of collisions also causes a longer delay. We can obtain from Figure 6c that OCA-MAC reduces the latency by about 50% and 30% compared to VeMAC and CAH-MAC, respectively. When the total number of nodes in the network increases, the choice of available cooperative nodes also increases. Therefore, the delays in CAH-MAC and OCA-MAC are both decreased. However, OCA-MAC always selects the optimal cooperative node that guarantees the minimum and stable latency with the increase of the number of nodes.

### 5.3. Influence of the Transmission Range

**Figure 7:**
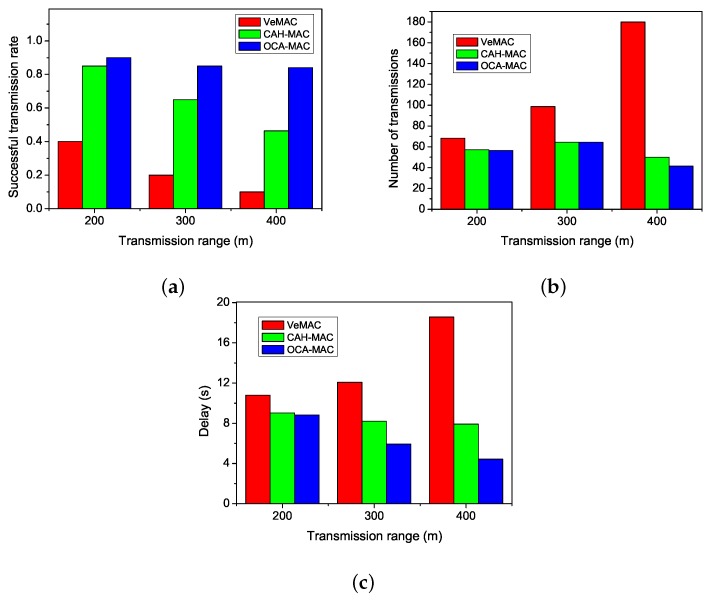
Influence of transmission range on the performance. (**a**) Successful transmission rate. (**b**) Number of transmissions. (**c**) Delay.

The experiment of varying the transmission range can reflect the characteristics of environments that have many interferences. These interferences can reduce the transmission range. Figure 7 compares the performance of OCA-MAC to that of VeMAC and CAH-MAC when the transmission range varies. It can be observed that OCA-MAC has a better performance than the other two MAC protocols.

Figure 7a indicates the relationship between successful transmission rate and transmission range. As the transmission range increases, the successful transmission rate of VeMAC evidently decreases. Compared to CAH-MAC, OCA-MAC improves the successful transmission rate by around 40% in the case of 400 m. When the transmission range increases, the number of available cooperative nodes also increases. OCA-MAC chooses the optimal cooperative nodes to forward data, but CAH-MAC only chooses cooperative nodes randomly. Therefore, OCA-MAC has a higher successful transmission rate than CAH-MAC.

Figure 7b shows the impact of transmission range on the number of transmissions. Because the transmission range becomes large, the probability of merging collisions is increasing. Obviously, VeMAC increases the number of transmissions sharply as the transmission range grows. The sending nodes generate a large number of retransmissions. In OCA-MAC and CAH-MAC, all nodes are synchronized and reserve their time slots. Therefore, the number of transmissions of CAH-MAC and OCA-MAC does not change significantly as the transmission range increases.

Figure 7c reveals the relationship between delay and transmission range. As the transmission range increases, the transmission delay of VeMAC also increases obviously. The growing number of retransmissions increases transmission latency. Although more cooperative nodes are available, CAH-MAC cannot guarantee to choose the best cooperative nodes to forward data. For this reason, the delay of CAH-MAC does not change apparently. However, OCA-MAC has more opportunities to choose the optimal cooperative nodes to forward data. Thus, the transmission delay of OCA-MAC decreases.

## 6. Conclusions

In VANETs, efficient MAC protocols have great impact on the performance of collaborative applications. In this paper, we proposed a new cooperative MAC protocol, called OCA-MAC, for vehicle-to-vehicle communication based on the CAH-MAC protocol. OCA-MAC optimizes selecting cooperative nodes to participate in transmission and exploits the free time slot to forward data. The node near the center position between the source node and the destination node has the highest probability to be the cooperative node. We also determined the number of cooperative nodes under different levels of channel quality through theory analysis. The experimental results showed that OCA-MAC effectively improves the network throughput compared to the existing protocols.

In this work, OCA-MAC only considers the situation that the moving speed among vehicles is relatively stable. It does not consider the dynamic topology changes among vehicles, which may influence the performance of cooperative transmission. The performance of OCA-MAC needs to be investigated in different mobility models, which represent various environments. In addition, the exchange of control messages results in much overhead. The method of controlling overhead should be also investigated in future work.

## Figures and Tables

**Table 1:** Basic simulation parameters.

Parameters	Values
Road length (L)	5000 m
Transmission range (r)	300 m
Vehicle density (β)	100 vehicles/km
Number of slots per frame (F)	60
